# Improved Survival and Retinal Function of Aging ZDF Rats in Long-Term, Uncontrolled Diabetes by BGP-15 Treatment

**DOI:** 10.3389/fphar.2021.650207

**Published:** 2021-04-16

**Authors:** Zita Wachal, Anna Szilágyi, Barbara Takács, Adrienn Mónika Szabó, Dániel Priksz, Mariann Bombicz, Judit Szilvássy, Béla Juhász, Zoltán Szilvássy, Balázs Varga

**Affiliations:** ^1^ Department of Pharmacology and Pharmacotherapy, Faculty of Medicine, University of Debrecen, Debrecen, Hungary; ^2^ Department of Oto‐Rhino‐Laryngology and Head and Neck Surgery, Faculty of Medicine, University of Debrecen, Debrecen, Hungary

**Keywords:** diabetes, survival, ZDF, retinopathy, ERG, BGP-15

## Abstract

Retinal complications of diabetes often lead to deterioration or even loss of vision. This hastens discovery of pharmacological agents able to counterbalance diabetic retinopathy. BGP-15, an emerging small molecule agent, was formerly proven by our workgroup to be retinoprotective on nonobese diabetic animals, Goto-Kakizaki rats. In the present study, we aimed to examine its long-term tolerability or incidental side effects on obese-prone Zucker diabetic fatty (ZDF) rats to further increase the rationale for a future human translation. To make terminal visual status comparable with our other investigations, we also carried out electroretinography (ERG) at the end of the experiment. Our study was started on 16-week-old ZDF rats and lasted for 52 weeks, while BGP was administered daily by gavage. During the 12 months of treatment, 100% of BGP-treated animals survived compared to the non-treated ZDF group, where 60% of the animals died, which was a statistically significant difference. Based on ERG results, BGP-15 was able to counterbalance visual deterioration of ZDF rats caused by long-term diabetes. Some moderate but significant changes were seen in OGTT results and some relationship to oxidative stress by the western blot method: BGP-15 was able to increase expression of HSP70 and decrease that of NFkB in eyes of rats. These were in concert with our previous observations of SIRT1 increment and MMP9 decrement in diabetic eyes by BGP. In summary, not only is BGP-15 not harmful in the long run but it is even able to reduce the related mortality and the serious consequences of diabetes. BGP-15 is an excellent candidate for future drug development against diabetic retinopathy.

## Introduction

Zucker diabetic fatty (ZDF) rat is the result of a mutation occurred in an originally obese/fatty strain of rats, namely the Zucker rats, as a consequence of which obese ZDF rats show glucose intolerance, hyperinsulinemia, and eventually type 2 diabetes due to the mutation-caused leptin resistance (Al-Awar et al., 2016). Although there are several other genetic rat models of diabetes, like Otsuka Long-Evans Tokushima fatty (OLETF), bioBreeding (BB), Wistar Bonn/Kobori (WBN/Kob), Spontaneously Diabetic Torii (SDT), and Goto-Kakizaki (GK), there is no best model to 100 percently mimic diabetes seen in humans (Olivares et al., 2017; Pandey and Dvorakova, 2020), not to mention the inavailability of some aforementioned animal subtypes in Central Europe from lab animal retailers. OLETF and BB, beside ZDF, are two more monogenic animal models, former harboring a G-protein mutation causing obesity and type II diabetes and latter being a type I diabetes model due to a mutation in lymphoid immune cells causing autoimmune response (Olivares et al., 2017). Formerly, we have conducted experiments with Goto-Kakizaki rats; however, this model did not develop obesity that is frequent in clinical setting (Akash et al., 2013). WBN/Kob and SDT rats, beside GK, belong to the group of polygenic diabetic retinopathy animal models, each presenting type II diabetes at different stages of life (Olivares et al., 2017). In case of ZDF rat, the process of development of type II diabetes and many of its characteristics, such as tendency to develop obesity, are identical to humans (Rees and Alcolado, 2005; Pandey and Dvorakova, 2020). Type 2 diabetes in humans also develops through similar course of symptoms starting from glucose intolerance through overt diabetes until serious consequences of diabetes, such as diabetic retinopathy (Rees and Alcolado, 2005; Heng et al., 2013).

Being a common microvascular complication of diabetes mellitus, diabetic retinopathy is one of the leading causes of blindness all over the world (Cheung et al., 2010). Clinically, diabetic retinopathy develops through 4 stages including pre-retinopathy, mild nonproliferative or background retinopathy, severe nonproliferative or pre-proliferative retinopathy, and finally proliferative retinopathy (Wong et al., 2016; Lechner et al., 2017). During nonproliferative retinopathy, funduscopic changes are diagnostic features including intraretinal microaneurysms and hemorrhages, lipoid exudates, cotton wool spots, and edemas (Wong et al., 2016). The consequence of proliferative retinopathy due to neovascularization can even be loss of vision (Wong et al., 2016; Wang and Lo, 2018). Functional identification of deteriorated vision of laboratory animals can be performed with the help of electroretinography (ERG) (Varga et al., 2011; Varga et al., 2013).

Background events leading to the aforementioned consequences of diabetes are proved to involve ischemic-reperfusion injuries to the retina (Calderon et al., 2017) based on or followed by changes of proteins such as heme oxygenase-1 (Varga et al., 2013), sirtuin 1 (SIRT1), or matrix metalloproteinase 9 (MMP9) (Wachal et al., 2020). Local ischemia increases vascular endothelial growth factor (VEGF) expression as well, which then leads to proliferative retinopathy with neovascularization (Lechner et al., 2017). Inflammatory pathways have also been detected to be involved in the complex mechanisms of diabetic retinopathy against which steroid and nonsteroid anti-inflammatory therapy can also be advisable (Wang and Lo, 2018), but surely new therapeutical approaches are welcome to treat or prevent vision decline.

Our workgroup has already made experiments on diabetic animals to counteract retinal damage with the help of retinoprotective agents, such as BGP-15, a promising future drug development target molecule (Pető et al., 2020). Being chemically related to propranolol, BGP was first invented for cardiovascular purposes: it was proved to be effective in ischemia-reperfusion-type injury in the heart (Szabados et al., 2000; Halmosi et al., 2001; Lampé et al., 2020). Diabetes itself is also related to ischemia-reperfusion injury, and as such, BGP-15 was tried out in diabetic cardiomyopathy as well by our workgroup to find that the agent is able to delay the onset of diastolic dysfunction in GK rats (Bombicz et al., 2019). Also related with ischemia-reperfusion damage and with inflammatory mediators, different pathologies came in focus of BGP-related research: this small molecule of hydroximic acid derivative was proved to be effective in nephro- (Racz et al., 2002), neuro- (Bardos et al., 2003), and myopathy (Sorensen et al., 2017) as well. In the different organs and in the different pathologies, naturally, BGP acted differently, but based on some general aspects of its effect—such as acting on heat shock proteins as an inducer, acting against superoxide and other reactive oxygen species (ROS) synthesis, inhibiting poly-(ADP-ribose)-polymerase (PARP), and increasing the number of mitochondria—the molecule was tried out on eyes of diabetic rats as well by our workgroup (Wachal et al., 2020). In that study, we found BGP to be able to overcome the retinal function deteriorating effect of type II diabetes in nonobese GK rats (Wachal et al., 2020).

As a basis for potential future drug developing purposes, the present study was conducted to assess any side effects of long-term BGP use: this experiment was aimed to find out if BGP-15 causes premature death in a long timescale, and if so, for what reason. Here, we used ZDF rats instead of GK as before, to experiment on obese-prone diabetic animals on the long-run: this has significant relevance in clinics, as most patients with type II diabetes have some overweight (Reinehr, 2005; Reinehr, 2013). As our article on the retinal effects of BGP-15 has already been published previously, it was not intended to perform more retinal-specific experiments, but electroretinography. Electroretinographical measurement is a relatively easy-to-perform and well-reproducible method that provides accurate information about the vision of the animals that allows easy comparison with our previous results. Since the animals had full uncontrolled diabetes during the one-year period, we definitely wanted to examine their eyes and compare them with our former results, but only electrophysiologically. Also, to lessen the insult that animals have to endure during this 1-year long study—besides daily gavage with the treatment material—we made measurements only at the end of the study.

## Materials and Methods

### Animals and Groups

Eight-week-old male ZDF rats (fa/fa) and their control, male lean rats (−/−), were purchased from Charles River Laboratories International, Inc. (Wilmington, MA, United States). Animals were housed and cared for based on rules acclaimed by the Institutional Animal Care Committee of University of Debrecen in accordance with international regulations (ARVO (Statement for the Use of Animals in Ophthalmic and Vision Research) and the NIH guidelines), and all methodical protocols were approved by this same committee. Animals had free access to water and rodent chow, Purina 5008 diet.

ZDF animals were randomly assigned into two groups: ZDF control group (ZDF) and BGP-15-treated group (BGP). A third group was formed from the lean animals (lean). *N* = 10 in each groups. From the age of 16 weeks, all animals received oral gavage (through an orogastric feeding tube) once daily throughout the whole study, which lasted for 52 weeks (1 year). The treated group received 10 mg/kg BGP-15 in methyl-cellulose mucilage, while animals in the lean and ZDF groups were gavaged with vehicle only. Dose was based on our former study (Wachal et al., 2020). BGP-15 was obtained from Sigma-Aldrich-Merck KGaA (Darmstadt, Germany).

### Calculation and Analyses of Survival Curve

During the long run of the study (52 weeks, i.e., 1 year), death of some of animals was observed and recorded. These data then were transferred to GraphPad Prism statistical analyzing program (version 7.0, GraphPad Software Inc., La Jolla, CA, United States) to create Kaplan–Meier survival curves. The curves were then analyzed using the Mantel–Cox test and Gehan–Breslow–Wilcoxon test.

### Oral Glucose Tolerance Test

The oral glucose tolerance test was carried out after a month of acclimatization (baseline values), then at the start of gavage period (start values), and then after 1, 6, and 12 months of gavage period.

After an all-night fasting, blood glucose was measured from tail vein using Accu-Chek glucose meter (Roche Diagnostics, Mannheim, Germany), and then, the animals were gavaged with glucose solution to administer 2 g of glucose per bodyweight kilograms (Sigma-Aldrich-Merck KGaA, Darmstadt, Germany). After 15, 30, 60, 90, and 120 min, blood glucose was repeatedly measured to obtain blood glucose level—time curves—so that the area under the curve (AUC) could be calculated using the fasting blood glucose values as starting concentrations (c0) at zero timepoint (t0). The following equation was used to calculate AUC, where “*n*” is the number of measuring timepoints

$$AUC=\left(\frac{c_{0}+c_{1}}{2}\right)*\left(t_{1}-t_{0}\right)+\ldots +\left(\frac{c_{n-1}+c_{n}}{2}\right)*\left(t_{n}-t_{n-1}\right)$$ (1)

### Electroretinography

Right after the final OGTT, animals were put to sleep with ketamine/xylazine combination (100/10 mg/kg) to carry out electroretinographical screening according to our formerly used method (Wachal et al., 2020). Pupils of both eyes were dilated with one drop of cyclopentolate (Humapent, Teva Ltd., Debrecen, Hungary), and then, a short funduscopic examination took place with a handheld ophthalmoscope (Heine Mini 2000 Ophthalmoscope, HEINE Optotechnik GmbH and Co. KG, Gilching, Germany) to confirm diabetic retinopathy—based on retinal status of ZDF rats with diabetic retinopathy characterized in detail in scientific literature (Kowluru et al., 2016; Mishra et al., 2016; Szabo et al., 2017). For the ERG measurement, five-needle electrodes were inserted into the animal to conduct the light-generated currents into an amplifier coupled with an analog-to-digital converter (Bridge Amp and PowerLab, ADInstruments, Sydney, Australia) to make waveforms visible on a computer using PowerLab Chart software (version 5.2.2, ADInstruments, Sydney, Australia). Two measuring electrodes were inserted lightly into the corneal surface (without perforating it) and two reference electrodes were stuck into the ears of the animal, while the general grounding electrode was stung into the skin of the glabella. Carbomer-based eye gel (Vidisic, BauschandLomb, Berlin, Germany) was used against dry out of the eyes and also as a contact gel.

**FIGURE 1:**
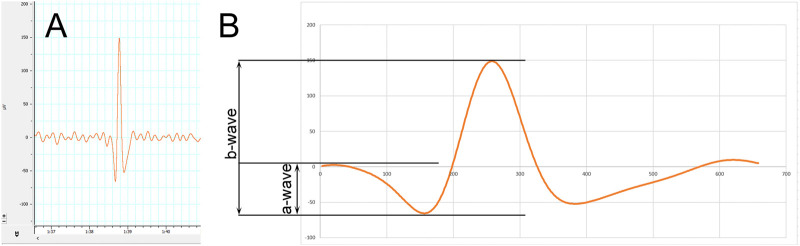
Methodology of electroretinography (ERG). **(A)** Electroretinogram showing a representative spike standing out from steady background noise. **(B)** A magnified spike showing the measured a‐ and b‐wave amplitudes.

Based on International Society for Clinical Electrophysiology of Vision (ISCEV) guidelines, electroretinographical measurements were carried out after a 20-min dark adaptation. Scotopic retinogram was recorded in darkness by illuminating the eyes of the animals with a stroboscope (20 cd/m^2^, 0.5 Hz) to elicit mixed rod and cone response. Recorded electroretinograms show definite, clearly identifiable spikes of electrical responses that emerge from background noise occurring consistently after and in rhythm with (i.e., with the same 0.5 Hz frequency as) the light stimulus of the stroboscope. As such, the highly positive peaks of each spike are the maximal values of b-waves, which are preceded by a-waves characterized by a negative peak as seen in other standard ERG systems (Varga et al., 2013). Amplitudes of b-waves were measured from the preceding negative maximum to the positive maximum, while that of a-waves were measured between the negative maximum and the preceding positive maximum (Figure 1). Electrical activity of both eyes of each animal were measured following 10 flash stimuli to create a common pool of recordings for each group, from which statistics were carried out as detailed in “*Statistical Analysis*” section. Former results of the authors with the same recording system assure that the used experimental method provides reproducible and reliable data on functioning of the retina, correlating with retinal integrity (Varga et al., 2011; Varga et al., 2013; Varga et al., 2017; Wachal et al., 2020).

### Western Blot

After the ERG, animals were gently exterminated by overdosing the anesthetic mixture. For further molecular biological analyses, eyes of the animals were extracted and homogenized with a knife homogenizer (IKA-WERKE ULTRA-TURRAX disperser, Staufen, Germany) in homogenization buffer on ice. The homogenization buffer contained the following: 25 mM Tris, 25 mM NaCl, 0.5 mM EDTA, protease inhibitor cocktail, and distilled water (all ingredients were bought from Sigma-Aldrich-Merck KGaA, Darmstadt, Germany). The homogenizate was then centrifuged (10,000 rpm, 20 min, 4°C) to separate the supernatant cytosolic fraction from the pellet. The pellet was further treated with homogenization buffer containing Triton X-100 tenzide as well (also from Sigma-Aldrich-Merck KGaA, Darmstadt, Germany), and after a thorough stirring and incubation separation of supernatant, nuclear fraction was done using centrifugation at 14,000 rpm, 10 min, 4°C. After measuring total protein concentration from each fraction (FLUOstar OPTIMA spectrophotometer, BMG Labtech, Ortenberg, Germany), samples were made ready for polyacrylamide gel electrophoresis by adding Laemmli sample buffer to them (Sigma-Aldrich-Merck KGaA, Darmstadt, Germany) and boil for 5 min. Proteins were separated on a 12% gel running for 100–120 min on 4 mA (Hoefer miniVe PAGE SE300 vertical electrophoretic and electrotransfer unit, Hoefer Inc., Holliston, MA, United States). Blotting of proteins to nitrocellulose membranes (GE Healthcare, Darmstadt, Germany) was done using the blot module of the aforementioned device (Hoefer miniVE SE300). After blocking with 3% BSA solution (Sigma-Aldrich-Merck KGaA, Darmstadt, Germany), proteins were incubated overnight with primer antibodies: anti-β-actin (Cat#A5316, Sigma-Aldrich-Merck KGaA, Darmstadt, Germany); anti-Histone H3 (Cat#701517, Thermo Fisher Scientific, Waltham, MA, United States); anti-HSP70 (SAB4200714, Sigma-Aldrich-Merck KGaA, Darmstadt, Germany); and anti-nuclear factor κ B (ab 16502, Abcam, Cambridge, United Kingdom). To visualize the sought proteins, horseradish peroxidase–linked secondary antibodies were used: anti-mouse antibody (Cat#A4416) and anti-rabbit antibody (Cat#A0545; both from Sigma-Aldrich-Merck KGaA, Darmstadt, Germany). For detection, LI-COR C-DiGit^®^ blot scanner (LI-COR Inc., Lincoln, NE, United States) and WesternBright™ enhanced chemiluminescent substrate (Advansta Inc., Menlo Park, CA, United States) were used. Analysis of three blots per group was carried out with ImageJ software (version 1.51, National Institutes of Health, Bethesda, MD, United States). Results were obtained by normalizing the blots to background and standardizing them to a housekeeping protein (beta-actin or Histone H3).

### Statistical Analysis

GraphPad Prism software (version 7.0, GraphPad Software Inc., La Jolla, CA, United States) was used for statistical analysis. Gaussian distribution was assessed with the Shapiro–Wilk normality test. Parametric data were tested with one-way analyses of variance (ANOVA), while nonparametric data with the Kruskal–Wallis test. Any comparison was considered significant, if probability values were lower than 0.05. The level of significance was indicated: *p* < 0.05; **p < 0.01; ***p < 0.001; and ****p < 0.0001. All data are presented as mean ± standard error of the mean (SEM).

## Results

### Survival Analysis Results

**FIGURE 2:**
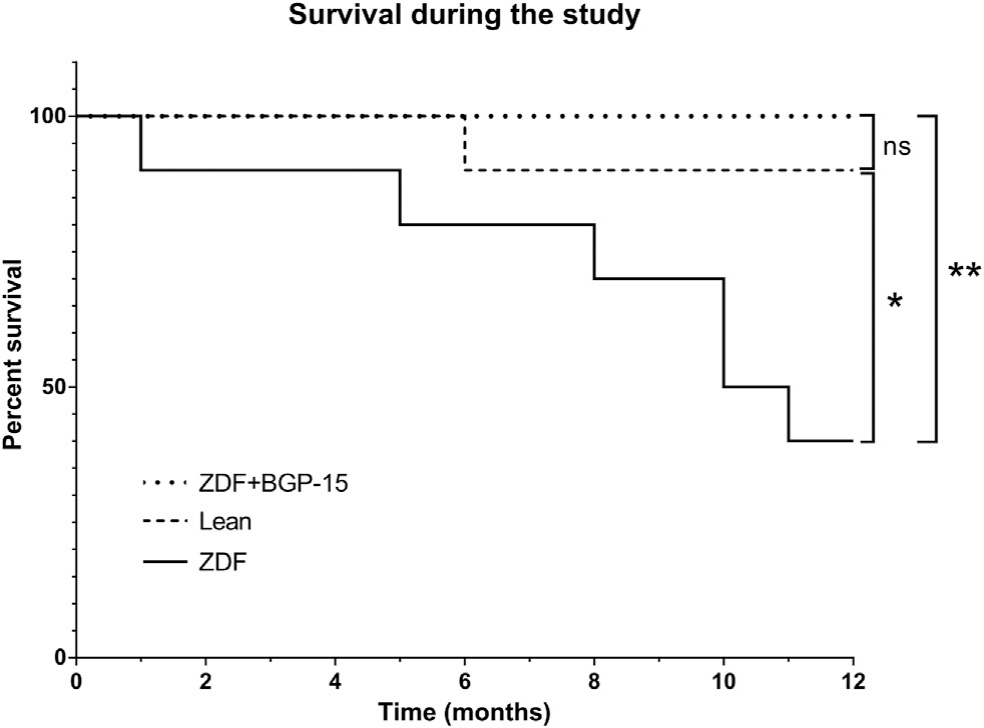
Survival of animals during the 12 months of the study. Sixty percent of the untreated ZDF animals died during the study, which is a significant difference compared to healthy lean animals (10% death) or BGP-treated ZDF group (100% survival) (*p < 0.05 and **p < 0.01, respectively). There was no significant difference between lean and ZDF + BGP-15 groups based on Mantel–Cox and Gehan–Breslow–Wilcoxon tests.

Analysis results regarding the survival of the BGP-treated animals are shown in Figure 2. During the 12 months of treatment, 100% of the animals of the BGP-treated group survived the deteriorating effects of diabetes as compared to the non-treated ZDF group, where 60% of the animals died, which proved to be a significant difference between the Kaplan–Meier survival curves of these groups (**p < 0.01). In the lean group, 90% of the healthy control animals reached the end of the study alive, which differed significantly from the 40% value of the non-treated ZDF group (*p < 0.05). There were no significant differences between BGP and lean groups based on Mantel–Cox and Gehan–Breslow–Wilcoxon tests.

Weight did not change differently between treated and untreated ZDF groups, although both differed significantly from lean values during the whole study (Supplementary Figure S1).

### OGTT Results

**FIGURE 3:**
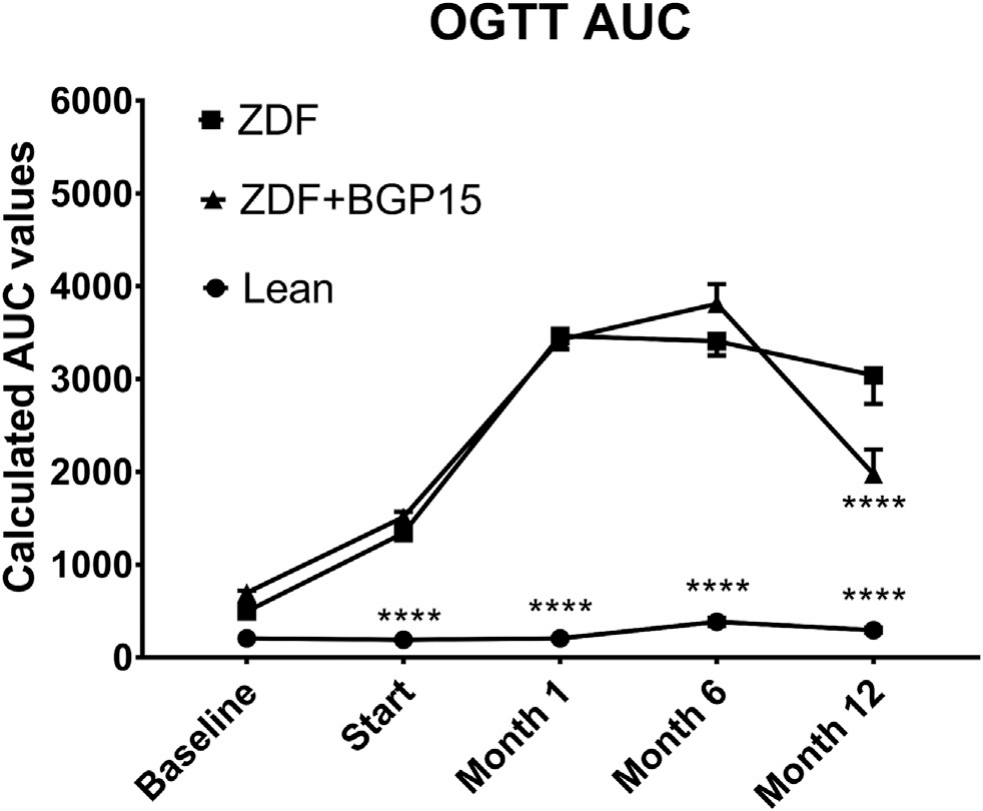
Oral glucose tolerance test (OGTT) area under curve (AUC) values during the study. Data are presented as group means. For a better visibility, standard error of the mean (SEM) of some data points are plotted only in one direction, or not plotted at all, if error bars were shorter than the height of the symbol. ****p < 0.0001 compared to ZDF group. Statistical analysis was done using GraphPad Prism: data were analyzed with the two-way analysis of variance (ANOVA) test.

Based on measurements of OGTTs (Figure 3), baseline area under the curve (AUC) values were similar in each group. At the start of the treatment period, however, diseased animal models already differed significantly from healthy control (lean) animals (1,338.873 ± 53.008 and 1,511.077 ± 56.820 vs. 190.211 ± 5.892 for ZDF and ZDF + BGP-15 vs. Lean groups, respectively, *p* < 0.0001 for both comparisons). This difference was maintained during the whole study. There were no significant differences, however, between the two diseased groups at this timepoint, or at 1-month or at 6-month timepoints. This was not the case in the 12-month timepoint, where AUC value of BGP-treated group was significantly different compared to untreated ZDF group (1976,027 ± 264,024 vs. 3040,019 ± 308,145, *p* < 0.0001).

**FIGURE 4:**
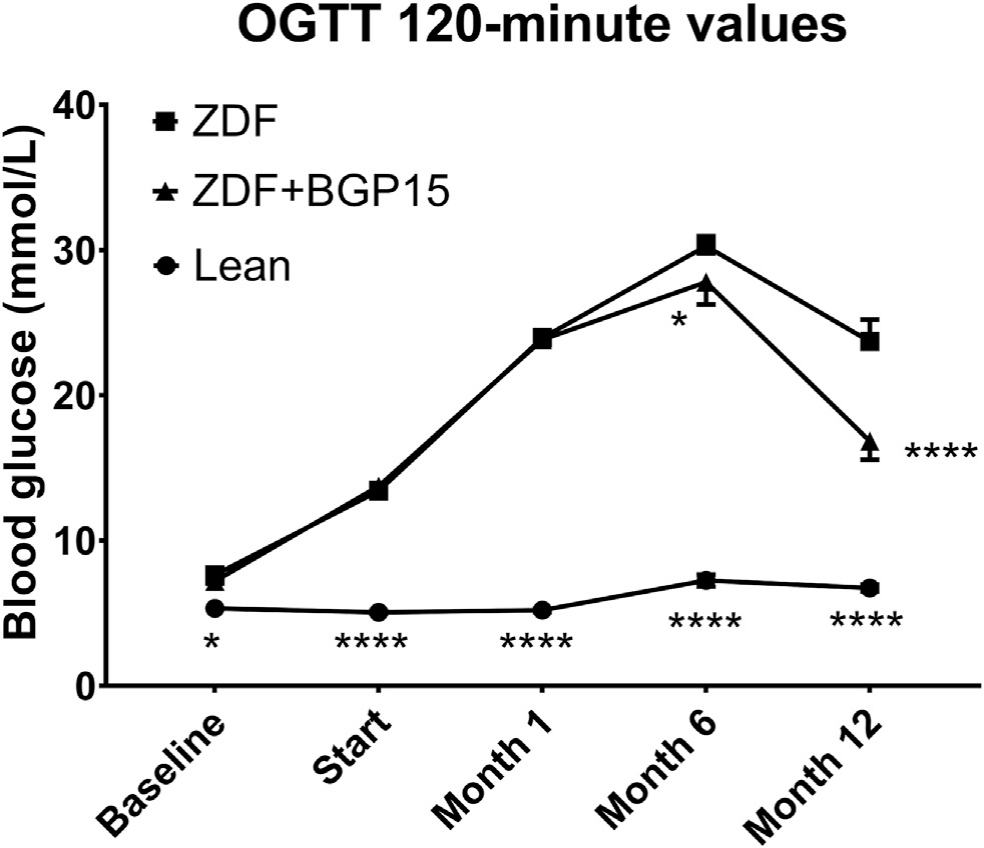
Oral glucose tolerance test (OGTT) 120-min values during the study. Data are presented as group means. For a better visibility, standard error of the mean (SEM) of some data points are plotted only in one direction, or not plotted at all, if error bars were shorter than the height of the symbol. *p < 0.05, ****p < 0.0001, both compared to ZDF group. Statistical analysis was done using GraphPad Prism: data were analyzed with the two-way analysis of variance (ANOVA) test.

If we take a look at OGTT 120-min values (Figure 4), the two-way ANOVA statistical analysis gave significant difference right at the baseline timepoint (7.630 ± 0.142 vs. 5.330 ± 0.078, for ZDF vs. lean, *p* < 0.05), which—similarly to AUC—was maintained during the whole study: mean values of lean group were under 7.5 mmol/L in all timepoints (5.330 ± 0.078, 5.050 ± 0.110, 5.200 ± 0.116, 7.244 ± 0.411 and 6.733 ± 0.236 mmol/L for baseline, Start, 1-month, 6-month and 12-month timepoints, respectively). Diseased groups showed higher values during the whole study; however, there proved to be some significant difference between treated and non-treated diseased group in 6-month and 12-month timepoints (30,275 ± 0.689 vs. 27,8 ± 1,548, for ZDF vs. ZDF + BGP15, *p* < 0.05 at 6-month timepoint, and 23,7 ± 1,522 vs. 16,84 ± 1,264 for ZDF vs. ZDF + BGP15, *p* < 0.0001 at 12-month timepoint).

Fasting blood glucose values (OGGT starting values) showed the same trend as 120-min values (Supplementary Figure S2).

### ERG Results

**FIGURE 5:**
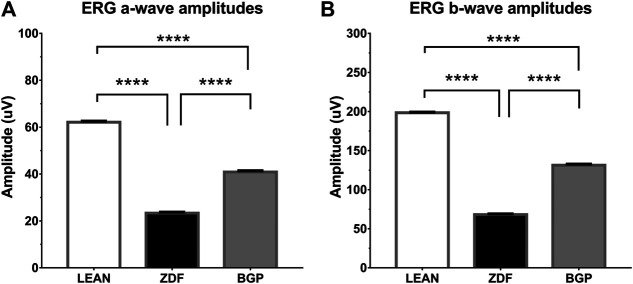
Results of electroretinography (ERG) measurements. **(A)** Group means of a-waves; **(B)** Group means of b-waves. All data are presented as group means ± standard error of the mean (SEM). ****p < 0.0001 Statistical analysis was done using GraphPad Prism: after estimation of Gaussian distribution with the Shapiro–Wilk normality test, data were either analyzed with the one-way analysis of variance (ANOVA) or the nonparametric Kruskal–Wallis test.

Electroretinographical results are shown on Figures 5A and B for a-waves and b-waves, respectively. According to ERG measurements, mean amplitudes of both a-waves and b-waves proved to be significantly different in all groups. The trends in case of a-waves and b-waves were the same: untreated ZDF animals produced significantly lower amplitude than healthy (lean) animals (in case of a-waves, these were 23.32 ± 0.4277 vs. 62.09 ± 0.4621, for ZDF and lean groups, respectively; in case of b-waves, these were 68.07 ± 0.9519 vs. 198.4 ± 0.7796, for ZDF and lean groups, respectively; *p* < 0.0001 in both comparisons), while BGP-treated ZDF animals produced significantly higher amplitudes than untreated ZDF group (40.88 ± 0.5149 for a-waves and 131.3 ± 1.408 for b-waves of BGP-treated ZDF animals; in both comparisons to corresponding ZDF values *p* < 0.0001).

Representative waveforms and all ERG amplitude values recorded are shown on Supplementary Figure S3.

### Western Blot Results

**FIGURE 6:**
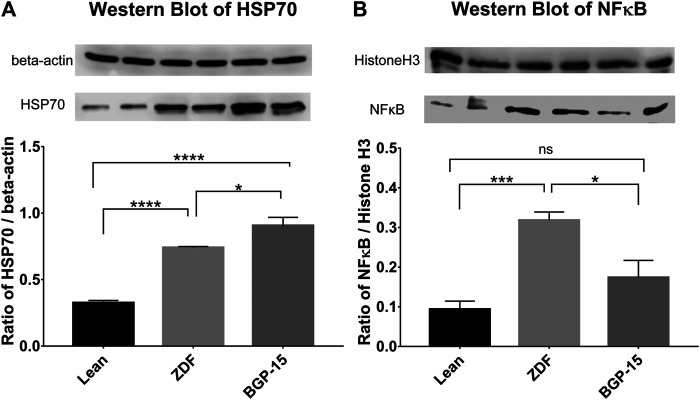
Western blot results. **(A)** Expression levels of heat shock protein 70 (HSP70) in the different groups. **(B)** Expression levels of nuclear factor kappa B (NFκB) in the different groups. All data are presented as group means ± standard error of the mean (SEM); *p < 0.05; ***p < 0.001; ****p < 0.0001; ns = nonsignificant. Statistical analysis was done using GraphPad Prism: after estimation of Gaussian distribution with the Shapiro–Wilk normality test, data were either analyzed with the one-way analysis of variance (ANOVA) or the nonparametric Kruskal–Wallis test.

According to western blot measurements, significant differences were observable between the expression levels of heat shock protein 70 (HSP70) and nuclear factor kappa B (NFκB) in the different animal groups (Figures 6A and B, respectively).

In case of HSP70 values, increased expression of this protein can be observed compared to healthy (lean) animals both in untreated and BGP-treated ZDF animals (0.7402 ± 0.0087 and 0.9059 ± 0.0621 vs. 0.3250 ± 0.0184 for ZDF and BGP vs. lean groups, respectively; *p* < 0.0001 in both comparisons). Treatment with BGP-15 further elevated the expression of HSP70, which also proved to be significant compared to mean value of untreated ZDF group (*p* < 0.05).

Similarly, NFκB expression showed elevation in both diabetic animal groups; however, this difference was only significant in case of ZDF vs. lean comparison (0.318 ± 0.0211 vs. 0.0933 ± 0.0210, *p* < 0.001), but not in BGP vs. lean comparison (0.1735 ± 0.0435 vs. 0.0933 ± 0.0210). BGP-treatment significantly decreased NFκB expression compared to untreated ZDF group (*p* < 0.05).

## Discussion

Eye-related complications of diabetes often lead to the most severe ophthalmological consequences such as deterioration or even loss of vision. Importance of diabetic retinopathy is further emphasized by the fact that it is one of the most common causes of blindness all over the world (Li et al., 2020). Therefore, it is of primary importance to discover new pharmacological agents able to counteract or at least alleviate or delay the deteriorating effects of diabetes. Although strict glycemic control can decrease and postpone complications (Fullerton et al., 2014), unfortunately, even current antidiabetic medications fail to effectively prevent the development of retinal impairment: despite adequate treatment, diabetic retinopathy develops after long time spent in diabetes (Hautala et al., 2014), which is further aggravated by age (Li et al., 2020) and comorbidities, such as hyperlipidemia (Kowluru et al., 2016).

Research of our workgroup is focused on complex pharmacodynamic screening of different active ingredients in diseased animal models, the purpose of which is to find new emerging drug candidates for later human drug development. One aspect of our research work is electroretinographical measurements to screen eye-related complications of diabetes keeping in mind potential human translational possibilities. Thus, it was pivotal to plan a long-term diabetic experiment for the present study to best model long time spent in diabetes. Our workgroup formerly carried out shorter tests with BGP-15, a hydroximic acid derivative on Goto-Kakizaki rats, a diseased animal model of type II diabetes mellitus (Wachal et al., 2020), and now we wanted to see BGP effects on the long run.

One of the most important achievements of the study is the result regarding the survival of the BGP-treated animals during the 12 months of the study (Figure 2). There has been few studies that operated with ZDF rats for a long time, for example, for 9 months in some other articles related to diabetic retinopathy, but they measured peripheral blood parameters or carried out histological analyses then (Mishra et al., 2016; Szabo et al., 2017). Cardiovascular function (Ferenczyova et al., 2020) and central nervous system protein changes (Nam et al., 2018) as well as endothelial dysfunction (Vessieres et al., 2013) have been assessed in 12-month-old ZDF animals, and also renal function in 12-month-old ZDFxSHHF hybrid rats (Tofovic et al., 2000), but to the best knowledge of the authors of the present article, there are no other articles comprising information about retinal function of ZDF rats diabetic for a whole year, 52 weeks. Similarly, the longest BGP treatment published so far was 12 weeks (3 months) in case of a muscular dystrophy model (Gehrig et al., 2012), and in case of our previous experiments on Goto-Kakizaki rats (Wachal et al., 2020). To our best knowledge, results of such long BGP treatment were not published so far. It is not unprecedented that a pharmacological treatment can extend survival in a diseased animal model (Li et al., 2019); however, the present article is the first publication that comprises results of a long-term BGP treatment to be so effective in a type II diabetic animal model, ZDF rats, maintained for 52 weeks.

Based on our OGTT results, BGP is not likely to exert the aforementioned prevention by settling glucose homeostasis; although, some significant differences were demonstrated in our present study (Figures 3, 4), instead the action of it might have some relationship to oxidative stress. Diabetes, even type II diabetes, is associated with higher risk of mortality (Coles et al., 2020), probably due to the accompanying oxidative stress (Rosales-Corral et al., 2015), which is proven to be among the causes of macro- and microcirculatory complications of diabetes (Madonna et al., 2017; Frisbee et al., 2019). Although at this point we have little information on the exact mechanism of action of BGP-15, it is at least a very telling result that no BGP-treated animals died during the 12 months spent in diabetes.

Functioning of the retina produces electric signals which are deteriorated in diabetes due to microcirculatory problems (Varga et al., 2017; Danis and Yang, 1993). Thus, the effect of BGP-15 seen in our electroretinographical results (Figure 5) might be connected to lessening of such microcirculatory complications. It is a remarkable novelty of our present study that BGP-15 is able to counteract the detrimental effect of long-term diabetes on the functioning of the retina of ZDF rats. Such retinal protection in diabetes is usually seen in case of neuropeptides, trophic factors, antioxidants, or in case of agents with anti-inflammatory action (Varga et al., 2011; Varga et al., 2013; Varga et al., 2017; Tomita et al., 2020). Although BGP is not a neuropeptide, nor a trophic factor, it might exert some antioxidant or anti-inflammatory properties on the retina as described before on non-retinal cell lines (Sumegi et al., 2017). In the background of the effects of BGP, different effector molecules are reported to be involved including HSP70 in diaphragmic muscle cells (Smuder et al., 2019), histone deacetylases in mouse endothelial fibroblast cells (Budzyński et al., 2017), RAC-alpha serine/threonine-protein kinase (AKT) in myocardial cells (Sarszegi et al., 2012), and Sirtuin 1 (SIRT1) in ocular cells of whole eye homogenate (Wachal et al., 2020). On the other hand, BGP-15 inhibits poly-ADP-ribose-poymerase 1 (PARP1) in myocardium (Szabados et al., 2000) and c-Jun N-terminal kinase (JNK)-pathway (Chung et al., 2008), or decreases the expression of MMP9 (Wachal et al., 2020). These molecular targets are parts of inflammatory and ischemic cascades and also occur in diabetic eyes, based on which we investigated HSP70 and NFkB involvement in the functional retinoprotective effects of BGP.

Our present results demonstrate for the first time that BGP-15 is able to increase expression of HSP70 in the eyes of ZDF rats. Seventy kDa heat shock proteins (HSP70) are ubiquitous chaperone molecules providing support for proper folding of many proteins, inhibiting their aggregation, and helping their removal if needed (Rosenzweig et al., 2019). HSP70 proteins are proven to play a protective role in several central nervous system diseases in which aggregation of aberrant or misfolded proteins initiate inflammatory processes followed by neuronal death (Turturici et al., 2011; Calsolaro and Edison, 2016). Due to this neuroprotective effect, protective role of HSP70 against eye diseases has emerged. Based on formerly demonstrated relationship with BGP-15, we analyzed HSP70 in our present study to find a significant increase in its expression (Figure 6A), which we believe to contribute to the functional retinoprotective effect of the treatment.

Advanced glycation end products (AGEs) and the consequential enhanced oxidative stress and low-level inflammation are also among the underlying causes of vascular complications of diabetes, and HSP70 is able to inhibit these inflammatory processes by sequestering NFkB, thereby reducing NFkB-induced iNOS expression and thus decreasing formation of reactive oxygen species (ROS) and peroxynitrites (Bellini et al., 2017). HSP70 also inhibits activation and translocation of NFkB and tumor necrosis factor alpha (TNFα) (Chung et al., 2008). These were the reasons this nuclear factor has become the target of our investigation. According to our western blot results in the present study, BGP-15 is able to decrease expression of NFkB in the diabetic eyes of ZDF rats, which is a novel result. This is consistent with our previous SIRT1-related findings (Wachal et al., 2020): SIRT1 inactivates NFkB and PARP1 physiologically; however, in diabetes, SIRT1 is downregulated leading to over-transactivation of detrimental genes in the diabetic eye, such as MMP9, an enzyme known to be involved in the development of mitochondrial damage in diabetic retinopathy (Kowluru et al., 2014a). It was formerly demonstrated that NFkB-dependent inflammation is an important trigger of endothelial insulin resistance and inhibition of this protein ameliorates transductional cascade of insulin and extends lifetime in mice (Paneni et al., 2013). Similarly to our experiments, carotenoids were proven to exert beneficial effects on the development of diabetic retinopathy by effectively decreasing NFkB levels in the eye of streptozocin-induced diabetic rat model (Kowluru et al., 2014b).

It is a rather general approach that the expression of HSP70 is induced in cellular stress, instead current articles study extra- and intracelluar HSP70 (eHSP72 and iHSP72) separately, former being a pro-inflammatory and latter being an anti-inflammatory inducible form of the 70 kDa HSP family (Hirsch and Heck, 2019). So, the overall picture is more complex, and since HSP70 and NFkB affect each other as mentioned in the previous paragraphs, a consolidated discussion is required. In diabetes, although the level of iHSP72 decreases, that of eHSP72 increases (Calderwood et al., 2016; Hirsch and Heck, 2019). Our hypothesis is that the summation of these changes is seen in the elevated total HSP70 level of diabetic ZDF animals in our present study. It has been proven by others that longer time spent in diabetes elevates eHSP72 more than shorter time (Nakhjavani et al., 2010), and that iHSP72 level in diabetes—without any treatment—is decreased (Kurucz et al., 2002; Di Naso et al., 2015), and it is also known that anti-inflammatory effect of iHSP72 is mainly mediated by NFkB-inhibition (Jones et al., 2011). Thus, presumably these are the reasons why total HSP70 level is high (compared to healthy group) together with an also high NFkB level in untreated ZDF group in our present, long experiment. Our hypothesis is that we have proven indirectly that this high level of HSP70 is due to increased eHSP72, which is corroborated by our results of high NFkB, showing a presumably high e/i ratio of the total HSP level. For this reason, some studies also recommended introduction of an H-index (e/i ratio) (Hirsch and Heck, 2019). BGP-treated animals are also diabetic, ZDF rats, so presumably they have the same high level of eHSP70, but with a difference. Here we see an even higher total HSP70 level, and moreover, a decrease in NFkB levels can also be observed in parallel, and as we already mentioned anti-inflammatory effect of iHSP72 is proven to be mainly mediated by NFkB-inhibition (Jones et al., 2011). Since NFkB levels were significantly decreased, while at the same time total HSP70 levels were elevated in our BGP-treated group compared to untreated ZDF animals, we believe this increase in total HSP70 is presumably due to an increase in the level of the NFkB-lowering, protective iHSP72. Thus, our second hypothesis is that we have indirectly proven that BGP-15 elevates the level of the beneficial iHSP72, which then shifts the e/i ratio to iHSP72 as it was able to exert a reduction in NFkB level, characteristic to iHSP72. In our present study, our two mutually reinforcing hypotheses were made possible by the fact that we isolated proteins not from separated retina but from whole eyeball samples, as seen in other studies (Jiang et al., 2010; Wang et al., 2010; Varga et al., 2017; Wachal et al., 2020). On the one hand, whole eyeball contains blood vessels as well—a reasonable and main location of eHSP72 (Mahmoud et al., 2018), as blood is also extracellular—and on the other hand, increased vascular permeability, characteristic to diabetic retinopathy, further elevates the interstitial appearance of eHSP in whole eye homogenate, contributing to the high levels of total HSP70 level in untreated ZDF group in our experiment. Moreover, vascular permeability is also a consequence of NFkB-activation due to hyperglycaemia and inflammation (Romeo et al., 2002). And in our present study, where BGP treatment decreased the level of NFkB, it presumably also decreased vascular permeability as well, thus the contribution of eHSP72 to the measured total HSP70 level presumably decreased as well further reinforcing our hypothesis, that the increment in total HSP70 level in treated animals is the consequence of the increase in the beneficial iHSP72. Limitation of our study is that we did not measure eHSP72 and iHSP72 separately, which means that their exact ratio cannot be determined. Thus, it cannot be ruled out that BGP-15 beside increasing iHSP may have decreased eHSP as well, in which case the e/i ratio may have become even more beneficial by BGP. So in the future to facilitate a more comprehensive understanding we consider it necessary to isolate retina for iHSP72-measurement and to measure eHSP72 from blood separately to further support our hypothesis, that BGP-15 is able to shift the ratio of eHSP72/iHSP72 in a beneficial way, in favor of iHSP72.

To draw a final conclusion based on our present results, not only is BGP-15 not harmful in the long run but it is even able to reduce the serious consequences of diabetes and at the same time the related mortality. By identifying its newer and newer molecular targets, we get closer to understanding the mechanism of effect of this special agent: inhibition of expression of NFkB and the increased level of HSP70 in the eye both contribute to its functional retinoprotective effects. In summary, BGP-15 is an excellent candidate for future antidiabetic drug development as it is able to counteract the functional deterioration of the retina due to long time spent in diabetes.

## Data Availability

The raw data supporting the conclusions of this article will be made available by the authors, without undue reservation.
