# Corrigendum: Improved survival and retinal function of aging ZDF rats in long-term, uncontrolled diabetes by BGP-15 treatment

**DOI:** 10.3389/fphar.2022.1034389

**Published:** 2022-09-16

**Authors:** Zita Wachal, Anna Szilágyi, Barbara Takács, Adrienn Mónika Szabó, Dániel Priksz, Mariann Bombicz, Judit Szilvássy, Béla Juhász, Zoltán Szilvássy, Balázs Varga

**Affiliations:** ^1^ Department of Pharmacology and Pharmacotherapy, Faculty of Medicine, University of Debrecen, Debrecen, Hungary; ^2^ Department of Oto-Rhino-Laryngology and Head and Neck Surgery, Faculty of Medicine, University of Debrecen, Debrecen, Hungary

**Keywords:** diabetes, survival, ZDf, retinopathy, erg, BGP-15

In the published article, there was an error in **affiliation 1**. Instead of “Department of Pharmacology and Pharmacotherapy, University of Debrecen, Debrecen, Hungary”, it should be “Department of Pharmacology and Pharmacotherapy, Faculty of Medicine, University of Debrecen, Debrecen, Hungary”.

The authors apologize for this error and state that this does not change the scientific conclusions of the article in any way. The original article has been updated.

