# Supplementary figures and images for: Improved Survival and Retinal Function of Aging ZDF Rats in Long-Term, Uncontrolled Diabetes by BGP-15 Treatment
Source: Front Pharmacol. 2021 Apr 16;12:650207. doi: 10.3389/fphar.2021.650207 (PMC8085539; doi:10.3389/fphar.2021.650207)

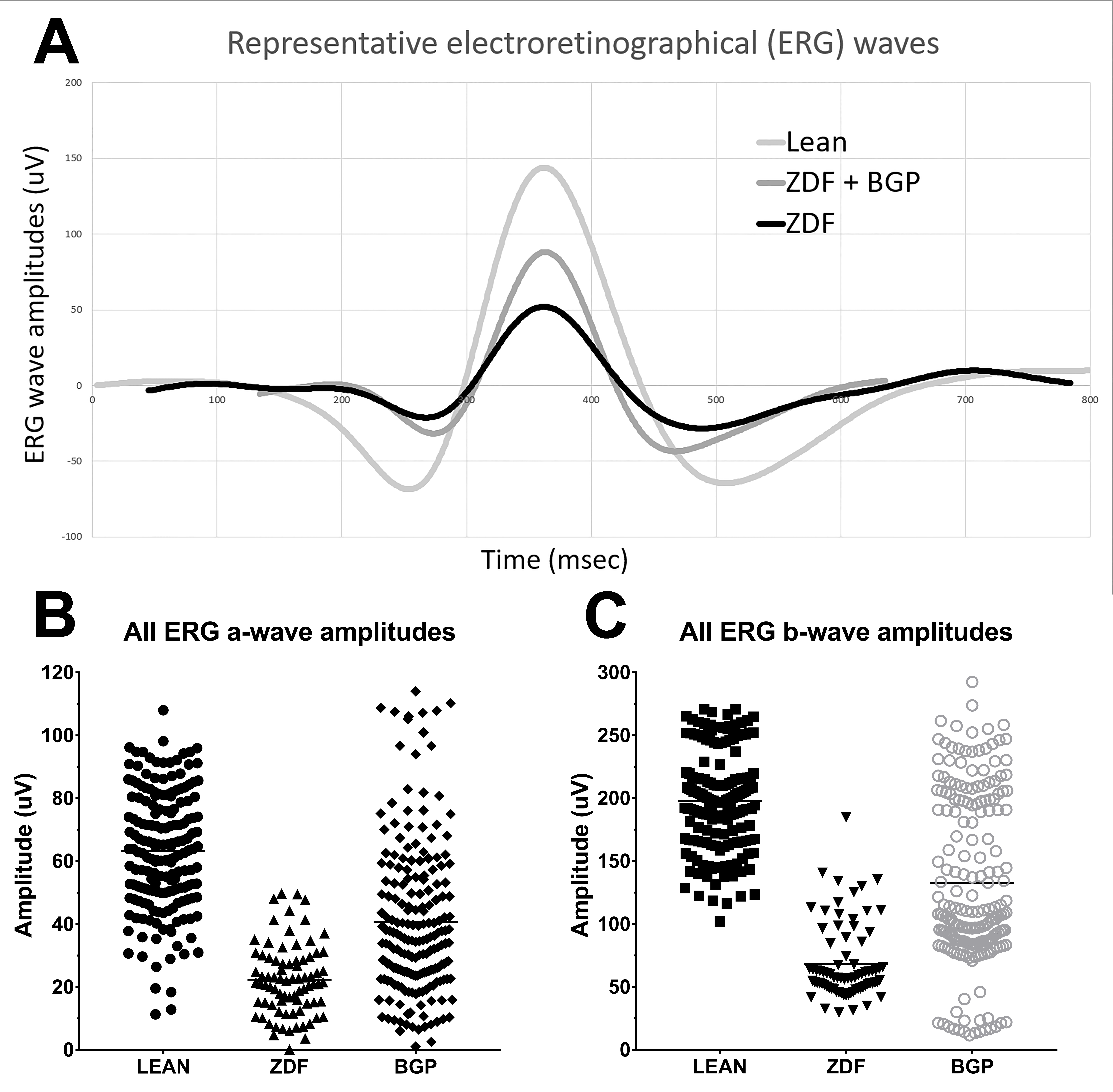

Supplement: Supplementary file 1 [file image3.tif]

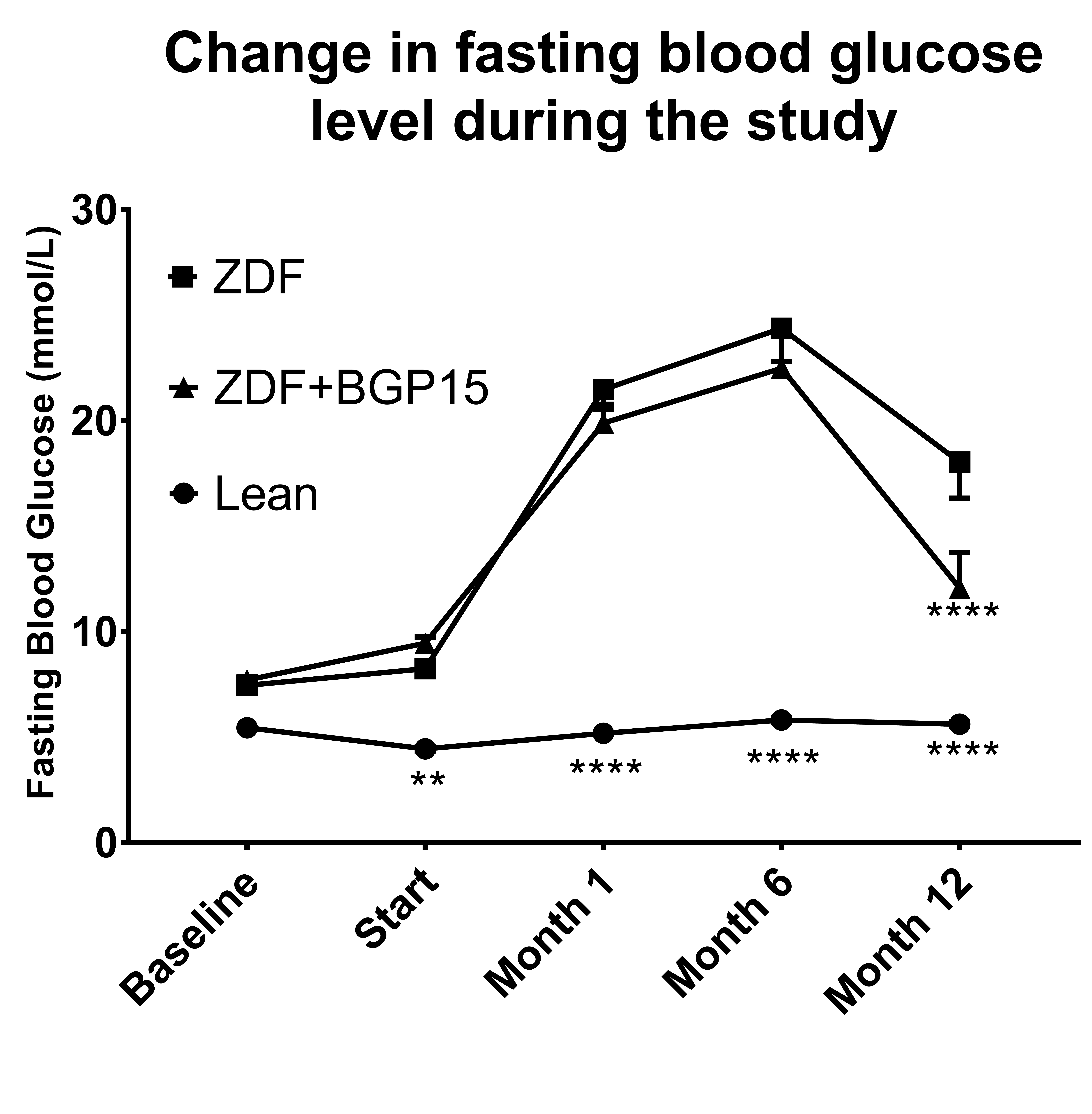

Supplement: Supplementary file 2 [file image2.tif]

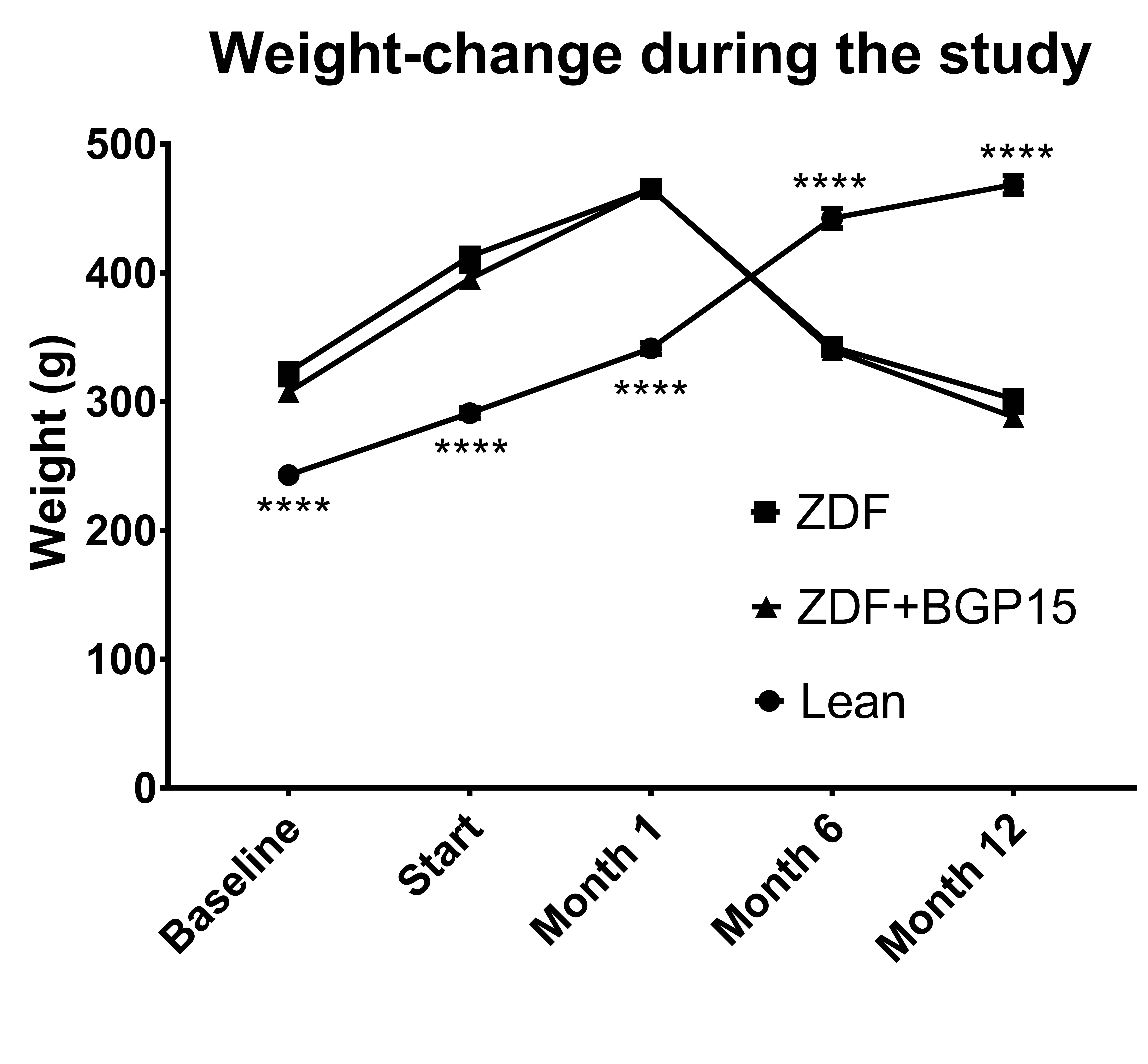

Supplement: Supplementary file 3 [file image1.tif]
